# Design & thermal modeling of solar panel module with embedded reconfigurable Air-Coil for micro-satellites

**DOI:** 10.1371/journal.pone.0199145

**Published:** 2018-07-31

**Authors:** Anwar Ali, Shoaib Ahmed Khan, Musaib Aleem Dildar, Haider Ali, Nasim Ullah

**Affiliations:** 1 Department of Electrical Technology, University of Technology (Shuhada-e-APS, UoT), Nowshera, Pakistan; 2 Department of Electrical Engineering, National University of Computers & Emerging Sciences, Peshawar, Pakistan; 3 Department of Electrical Engineering, Abasyn University, Peshawar, Pakistan; University of California Merced, UNITED STATES

## Abstract

Spacecrafts need to maneuver their solar panels towards the sun and antennas towards the ground station for maximum solar power harvesting and communication with the ground station. For tracking purpose, usually magnetorquer rods, reaction wheels and permanent magnets are used, but they are heavier, expensive, and occupy extra space on the spacecraft. Keeping in mind the dimension, budget and mass constraints of small satellites, a system compatible with small satellite is worth consideration. Consequently, this paper focuses on designing and analyzing a solar panel module with embedded Air-Coil. Such an Air-Coil is an innovative idea for the replacement of heavier, bulky and expensive attitude control systems. The proposed Air-Coil is integrated in the internal layers of an eight layers solar panel PCB module. Complete degradation analyses of the solar panel have been done to ensure that it will meet the satellite power requirements at BOL (beginning of life) and EOL (end of life). The proposed embedded Air-Coil has been analyzed for the generated magnetic moment, resultant torque, power consumption and temperature increase of the complete solar panel unit. A steady state thermal model is proposed to measure the thermal resistance between top and bottom layers of the solar panel module, which gives an idea about the heat trapped inside the solar panel module. The designed embedded Air-Coil is fully reconfigurable where coil in each layer can be operated as a separate coil. The four coils in different layers can be attached or detached through switches in different configurations i.e. single coil, four in series, four in parallel, and their hybrid combinations. The analyses of power consumption, heat dissipation, temperature rise, magnetic moment and torque generation by different configurations of the designed Air-Coil have been performed. The generated magnetic moment is very high i.e. around 12Am^2^, which is enough to rotate a microsatellite by 90° in 200s. The efficacy of the proposed module is significantly higher than the already available systems with respect to mass, price, power dissipation, heat generation, and dimension.

## 1. Introduction

Many academic institutions and space agencies around the globe are working on the design and development of small satellite projects [1, 2], because of its low price and short development time. Small satellites are categorized into Pico, Nano and Micro-Satellites with respect to their mass and size. Microsatellites mass is varying between 10kg and 100kg [3] and are launched into low earth orbits (LEO). The purpose of this work is to design and analyze a fully modular solar panel with embedded Air-Coil according to the required microsatellite standards.

The idea of microsatellite is related to CubeSat which is initiated in 1999 at Stanford University and California Polytechnic State University (Cal Poly). CubeSat is basically a small satellites of a few kilograms (usually no more than 1.33 kg) and has a standard set of dimensions usually 10×10×10 *cm*^3^ [4]. Studying a small satellite is very important for the academic institutions, as it provides hands on experience for the students to investigate and research space and upper atmosphere. Usually, commercial off the shelf (COTS) components are used for the development of small satellites which are inexpensive and available in the local market. Therefore, small satellites are the focus of research and motivating in academic institutions.

Microsatellite is powered by the solar panel mounted on its outside periphery, which is designed according to the power requirement of the satellite subsystems. These Satellites have directional components which should be directed and positioned at specific inertial frame of axis. Antennas should be directed at ground station and solar panels towards the sun, which need an efficient and stable actuation system. For this purpose, usually magnetic rods, reaction wheels, and magnetic rods are used. Permanent magnets are inexpensive, designing is simple and have no power requirement, but they have poor pointing accuracy and insufficient pointing direction. Magnetic rods and reaction wheels have the best pointing accuracy and can position the satellite in any direction but the problems with these systems are their heavier mass, large size and they are very expensive which make them unsuited with microsatellites [5]. Consequently, innovative embedded Air-Coil is required for the stabilization and rotational purposes of the microsatellite. Air-Coil is a preferable design in terms of pointing accuracy, cost, mass, size, and mass for microsatellite [6]. They are almost massless (PCB traces), consume minimum possible power, and generate torque according to the design requirements.

**Fig 1:**
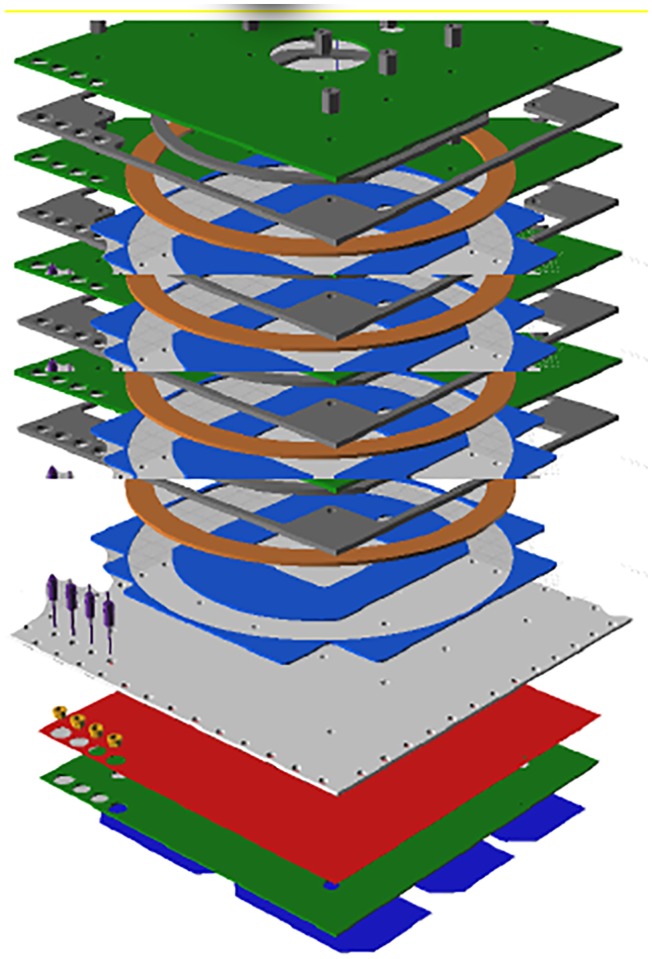
Internal layer-wise view of the solar panel PCB.

The solar panel module (SPM) with embedded Air-Coil is being designed for the SUPARCO satellite PNSS-1 (Pakistan national student’s satellite-1) [7]. SUPARCO is Pakistan national space agency, which has formulated a Pakistan national student satellite program (PNSSP). This program aims to involve local universities, industry and SUPARCO in collaboration for the design and development of small satellite. PNSS-1 is the first of this series of small satellites. Different universities submitted their proposals for the subsystem design and development of PNSS-1. Solar panel module with embedded Air-Coil was proposed and got accepted. According to the proposal, solar panel will be an eight layers PCB, having solar cells on top layer, embedded Air-Coil in the four internal layers (2^nd^, 3^rd^, 4^th^ & 5^th^), ground planes in 6^th^ & 7^th^ layers and Air-Coil driver with different sensors on the bottom layer. Fig 1 shows the enlarged layer-wise view of the proposed solar panel PCB.

Air-coil is a current carrying electromagnetic coil producing magnetic field lines that intermingles with the earth’s magnetic field and orients the satellite in the desired direction according to the motor action principle. In LEO orbit, the earth magnetic field strength varies between ±0.4 Gauss. When current ‘*I*’ runs through the Air-Coil with ‘*N*’ number of turns and with a cross sectional area ‘*S*’, a magnetic moment $\vec{D}$ is produced which is given by (1) [8,9];

$$\vec{D}=N.S.I\;\hat{n}$$ (1)

**Fig 2:**
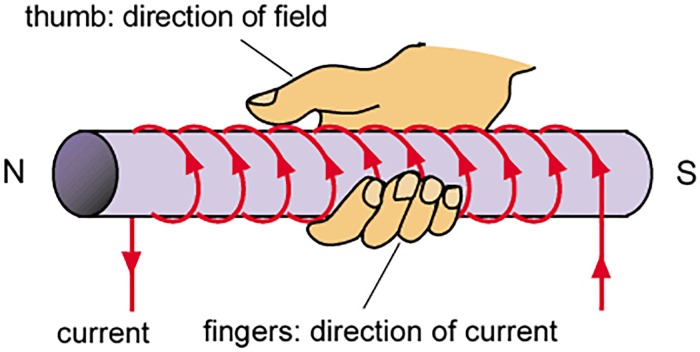
Magnetic moment direction given by right hand rule.

The direction of $\vec{D}$ is represented by the right hand rule which illustrates that grip the solenoid in right hand such that the curly fingers point in the path of current then the thumb in the axis of solenoid provides the direction of magnetic moment $\vec{D}$ as shown in Fig 2 [10].

When this coil is employed in a magnetic field, a torque (*τ*) is applied upon it which is given by (2) [8, 9];

$$\vec{\tau }=\vec{D}\times \vec{B}=DBsin\theta \hat{\;n}$$ (2)

**Fig 3:**
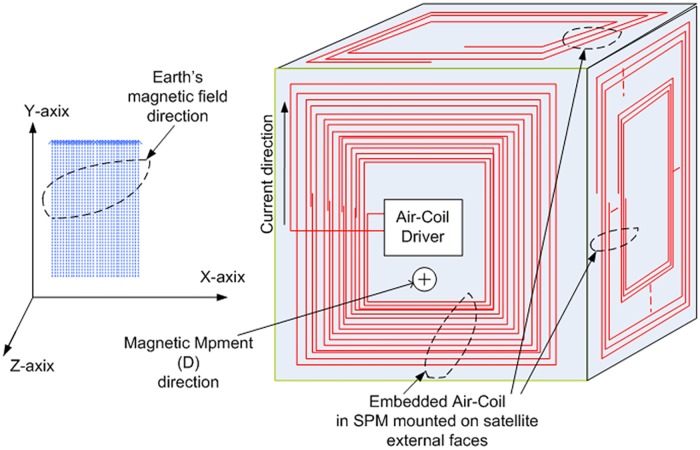
Earth magnetic field interaction with Air-Coil magnetic moment.

Where *θ* is the angle between $\vec{D}$ and $\vec{B}$. Fig 2 explains interaction of magnetic moment ($\vec{D}$) produced by the Air-Coil, the earth magnetic field ($\vec{B}$) and the direction of the resultant torque ($\vec{\tau }$) exerted on the system. In Fig 3, the cubic box represents a microsatellite with six external faces mounted on the solar panels having embedded Air-Coils. When current flows in clock wise direction through the Air-Coil, magnetic moment $\vec{D}$ is generated. According to right hand rule, the path of $\vec{D}$ is towards the paper (i.e. in the negative Z-axis). In case of the earth magnetic field in Y-axis, a torque is exerted on the satellite in X-direction which is given by Fleming’s left hand rule. The resultant torque rotates satellite in anti-clock wise direction. As all the six faces have solar panels with embedded Air-Coils, the satellite can be orientated in any direction by energizing the respective Air-Coil.

The embedded Air-Coil generates heat inside the solar panel PCB, therefore detailed thermal analysis is required for the embedded Air-Coil and the mounted solar cells. Thermal analyses have been done in great detail to determine the thermal aspects.

The paper is ordered according to the following structure. Section 2 provides details of solar panel design, section 3 explains the design of Air-Coil, section 4 thermally analyze the system, section 5 presents the comparative analysis among different combinations of the Air-Coil, section 6 discusses thermal modeling of the solar panel unit and section 7 concludes the paper.

## 2. Solar panel design

Solar panel module (SPM) design is the most significant step in the development of a microsatellite. Selecting the string size which is too small for rated solar panel design will sacrifice efficiency. On the contrary, selecting an oversized string will damage the converter and the equipment associated with it [11]. Photovoltaic (PV) cells can be attached in any arrangement i.e. series or parallel. Series contacts increase the voltage of the string and retain the current constant while parallel arrangements increase the current of the string and voltage is constant. The association between ambient temperature and string voltage must be taken into deliberation when computing string size [12]. Ambient temperature and PV array output power are inversely related with each other. Low temperatures will result in high output power while high temperatures will decrease the power. The effects of the temperature gradients between minimum and maximum limits cannot be ignored because there are high temperature variation eclipses during the full sun conditions. Therefore, proper and accurate power analysis is essential to compute the power ratings for efficient SPM design [13].

**Fig 4:**
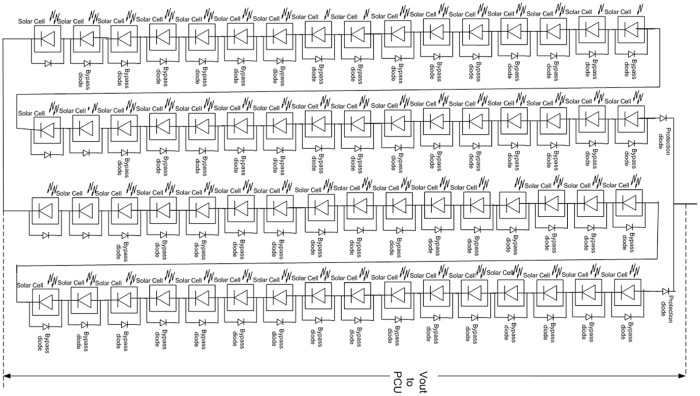
SPM schematic.

**Fig 5:**
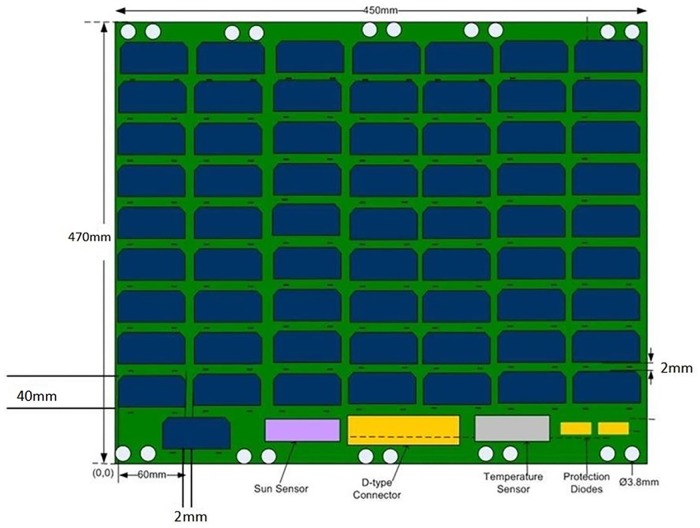
SPM PCB layout.

PV cells array is less efficient than the sum of individual cells because of the manufacturing and environmental issues, collectively called as degradation factor [14]. The degradation factor is taken into account for the solar panel design calculations. For minimum voltage, maximum operating temperature must be considered, because as the temperature increases above the standard test conditions (STC) i.e. 25°C, the voltage decreases corresponding to per degree rise in temperature. For calculating upper voltage limit, minimum operating temperature is considered because, as discussed earlier, when the temperature decreases below STC, the voltage increases corresponding to per degree decrease in temperature [15]. According to SUPARCO requirements, the SPM should be capable to generate power 40W~80W with voltage range 50V to 85V. The solar panel dimensions are 470×450mm^2^, which will not only accommodate enough PV cells to generate the required power but also have temperature and sun sensors. Keeping in mind the SPM intended life span, triple junctions GaAs (CESI manufactured) space qualified solar cells are used in the design [16]. To meet the power and voltage requirements, 64 solar cells were used. They are divided into two strings each having 32 cells in series along with 64 bypass and two protection diodes. The temperature in LEO varies between -40°C and 80°C. The maximum and minimum numbers of solar cells, voltage, current, and power ratings have been calculated after applying degradation analysis [14]. The parameters in Table 1 show that the power ratings are inside the design constraints [7]. Fig 4 shows schematic of SPM while Fig 5 shows the SPM PCB layout.

**Table 1:** SPM performance parameters.

SPU Parameters	value
Minimum voltage of single Solar cell after degradation, V_min_	1.73V
Maximum voltage of Solar cell after degradation, V_max_	2.62V
Minimum number of solar cells required	23
Maximum number of solar cells Required	32
Minimum voltage of Solar panel after degradation, V_min_	55.42V
Maximum voltage of Solar panel after degradation, V_max_	83.84V
Average voltage of Solar panel, V_avg_	69.63*V*
Short circuit current, I_sc_	0.42A
Short circuit current after degradation, I_sc_	0.36A
Minimum generated power of Solar cell, P_min_	0.62W
Maximum generated power of Solar cell, P_max_	0.94W
Average generated power of Solar cell, P_avg_	0.78W
Minimum generated power of Solar Panel, P_min_	39.68W
Maximum generated power of Solar Panel, P_max_	60.36W
Average generated Power of Solar panel, P_avg_	50.02W
Number of Solar cells in each string	32
Number of Solar cells in two strings	64

## 3. Embedded Air-Coil design description

**Fig 6:**
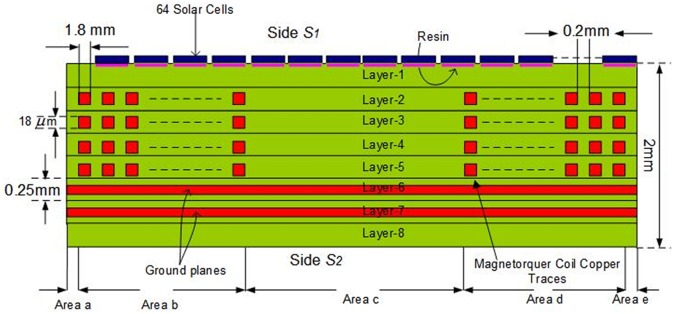
Cross sectional view of solar panel unit module.

**Fig 7:**
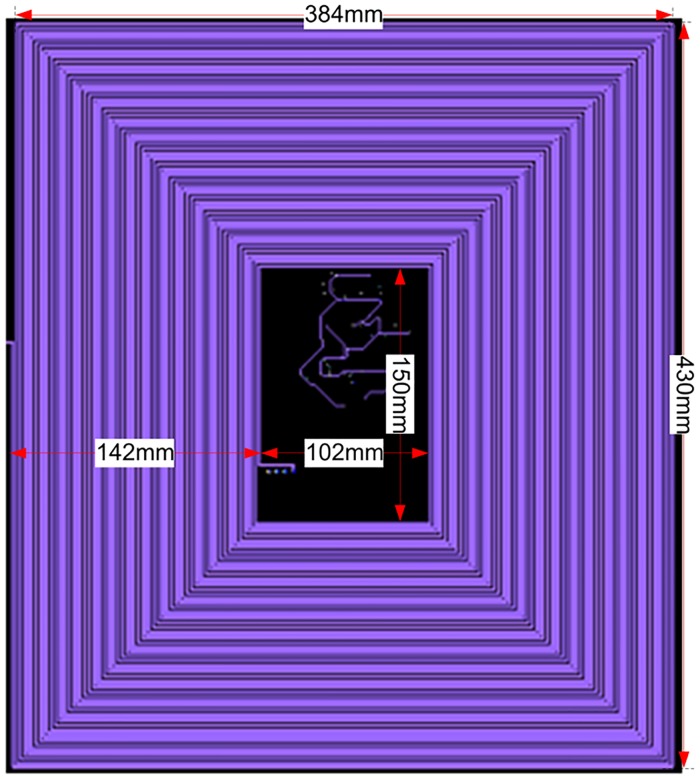
Air-Coil design.

The concept is to integrate the Air-Coil inside the solar panel four internal layers i.e. 2^nd^, 3^rd^, 4^th^ & 5^th^ layers. According to PNSS-1 requirements, the Air-Coil should be able to provide a magnetic moment of at least 6 Am^2^. To generate the required magnetic moment, 65 turns of embedded Air-Coil copper traces are mandatory in every layer and a total of 325 turns in four inner layers. Each coil trace has 1.8mm width and 18um thickness. The spacing between two attached traces is 0.25mm. The area occupied by a single turn out of all 65 turns is different from each other. For magnetic moment calculation (as given in (1)), an average cross sectional area has been fixed. In Fig 6, the external side ‘B’ has a length of 430mm and the corresponding internal side length is 150mm resulting in an average length of 290mm. Similarly, external side ‘A’ is 384mm and internal side is 102mm that gives an average length of 243mm. The average length of the single turn is 1066mm and 65 turns have a total length of 69m. Length, trace area (thickness and width) and the total number of turns change the overall impendence, magnetic moment, power consumption, and generated heat of the Air-Coil. Internal view of the PCB is shown in Fig 6 while the embedded air coil along with its dimensions is shown in Fig 7. The key motivation of the Air-Coil design is not only to make it compatible with the SPM dimensions but also be lighter and capable to generate the intended magnetic moment [17]. The main objective of the design is to make it reconfigurable and compatible with the SPM dimensions. Air-Coil can be designed in different shapes i.e. square, circular, or rectangular. In case of the PNSS-1 SPM, the most suitable shape is rectangular [18, 19].

The Air-Coil module is composed of embedded Air-Coil and its driver circuitry mounted on layer 8 of the SPM. Air-Coil driver receives 18V input power from the power distribution bus (PDB). It communicates with on-board computer (OBC) through redundant CAN buses. Current, voltage and temperature sensors are employed at different points of the driver for housekeeping purposes. The driver is monitored and controlled by PIC microcontroller based on CMOS devices which are prone to latch-up events in the space radioactive environment [20]. Latch up protection system is also designed to keep the unit from hazardous situations.

### 3.1. Design re-configurability

**Fig 8:**
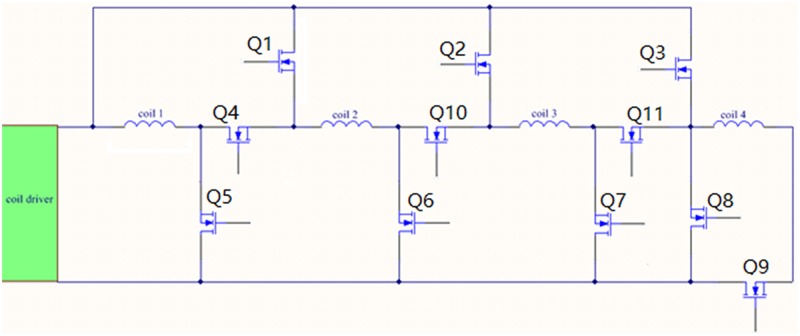
Four embedded Air-Coils attached through switched for design re-configurability.

The main objective of this design is to make the Air-Coil fully reconfigurable and compatible with the microsatellite dimensions. Each coil in the four internal layers (coil 1, coil 2, coil 3 & coil 4) can be used as individual coil or can be attached as four in series, four in parallel and in any hybrid combination for effective management of directionality and controlling intruding factors as shown in Fig 8. These coils can be attached or detached through transistor switches (Q1 ~ Q11) by a control signal from the processor. By changing the ON/OFF status of these switches, one can use the embedded Air-Coil in any configuration. The design reconfigurability gives options for generating precise magnetic moment and subsequent torque, managing power consumption and limiting heat dissipation. The ON/OFF combination of switches and specific coils configuration is shown in Table 2.

**Table 2:** Switches combination for specific configuration of coils.

S. No.	Air-Coil Configuration	ON Switches	OFF Switches
1	Single Coil	Q5	Q1, Q2, Q3, Q4, Q6, Q7, Q8, 9Q, Q10, Q11
2	Four coils in series	Q4, Q10, Q11, Q9	Q1, Q2, Q3, Q5, Q6, Q7, Q8
3	Four coils in parallel	Q1, Q2, Q3, Q5, Q6, Q7, Q9	Q4, Q10, Q11, Q8
2	Hybrid Combination	Q4, Q6, Q2, Q11, Q9	Q1, Q3, Q5, Q7, Q8, Q10

### 3.2. Applied voltage versus torque generated

**Fig 9:**
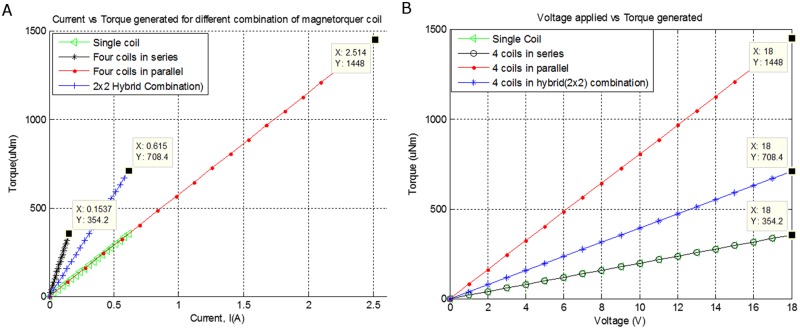
(a). Current versus generated torque. (b). Voltage versus generated torque in presence of 0.5-Gauss Earth magnetic field.

Air-Coil driver controls the applied voltage and manages the amount of current, the resultant magnetic moment, torque generated and heat dissipated through different possible configurations of the embedded Air-Coils. Applied voltage versus torque and resultant current versus torque are given in Fig 9(a) and 9(b) respectively, which shows different parameters of interest for various configurations of the Air-Coil. At 18V applied voltage a current of 2.5A flows through 4 coils in parallel which generates 1448 uNm torque. At the same applied voltage i.e. 18V, 2x2 hybrid combination draws 0.6A current and produces 708 uNm torque. In case of single coil and 4 coils connected in series, the produced torque is the same i.e. 354 uNm, while the current drawn by single coil is 4 times greater than the 4 coils in series.

### 3.3. Microsatellite rotation methodology

The satellite angular speed (*ω*) is dependent on the torque (*τ*) applied and the satellite’s inertial moment (*J*). Suppose the satellite has to cover an angular distance ‘*φ*’, a specific torque ‘*τ*’ for a definite time (0 → *T/2*) is required to increase the satellite angular speed (*ω*), linearly. In order to cease the satellite angular motion, the same magnitude and opposite direction torque (*−τ*) for the same duration (*T/2 → T*) must be applied. The reverse torque halts the satellite motion after covering the desired distance (*φ*). According to Newton’s second law of rotational motion,

τ=Jω (3)

ω=∫0TτJdt=∫0T2τmaxJdt+∫T2T−τmaxJdt=(τmax.tJ|0T2;τmax.(T−t)J|T2T) (4)

The following equation demonstrates the angular position (*φ*);

φ=∫0Tω.dt=(τmax.t22J|0T2;τmax.TJ(t−t22T−T4)|T2T) (5)

$$\varphi =\omega {\left(\frac{T}{2}\right)}^{2}+\omega {\left(\frac{T}{2}\right)}^{2}=\frac{\tau }{2J}T^{2}$$

The time *T* required for the Air-Coil to revolve the satellite through a definite angular distance *φ* can be found by (6);

$$T=\sqrt{\frac{2J\varphi }{\tau }}$$ (6)

**Fig 10:**
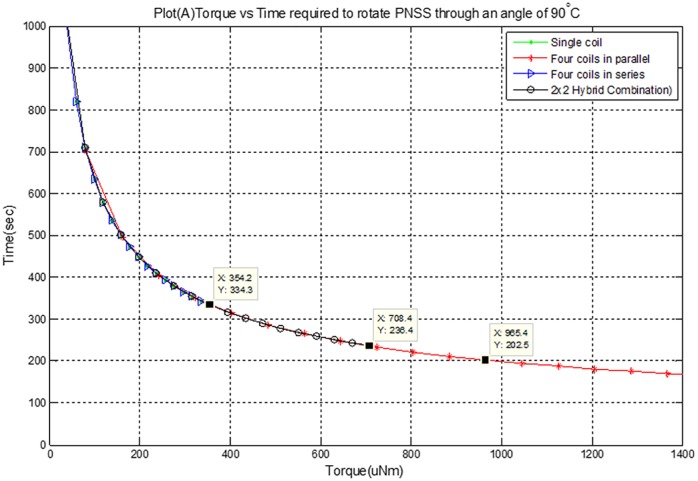
Torque versus time for different configuration of coils.

Eq (6) shows that torque applied and time required to revolve the satellite are inversely related. Fig 10 shows the torque produced and the equivalent time to revolve the microsatellite at an angle of 90° by energizing various arrangements of the coils. The value of *J* for the required microsatellite to be designed is 6.23kgm^2^. The torque generated by 4 coils in parallel is 944uNm, which takes 204s to rotate the satellite through an angle of 90°. In case of single and 4 coils connected in series, its generated torque is 354uNm, which takes 334s to revolve the satellite through an angle of 90°. In case of 2×2 hybrid combination, torque produced is 708uNm, which needs 235s for 90° rotation.

## 4. Thermal analysis equations

Thermal analysis has great significance in the designing of an embedded Air-Coil [21]. When electrical power is applied to the embedded Air-Coil, heat power is dissipated which increases overall temperature of the solar panel unit [22]. This rise in temperature should be analyzed to ensure manageability of preferred temperature within definite limits and to avoid failures which may lead to system permanent loss.

Thermal analysis provides details about thermal power consumption and the resultant temperature variations of the solar panel unit. Power consumption is organized by checking the amount of current that flows through the Air-Coils. Current flow also confines the amount of magnetic moment generated and thus temperature and magnetic moment are inter-related quantities. Greater magnetic moment can be attained at the cost of higher solar panel unit temperature.

**Fig 11:**
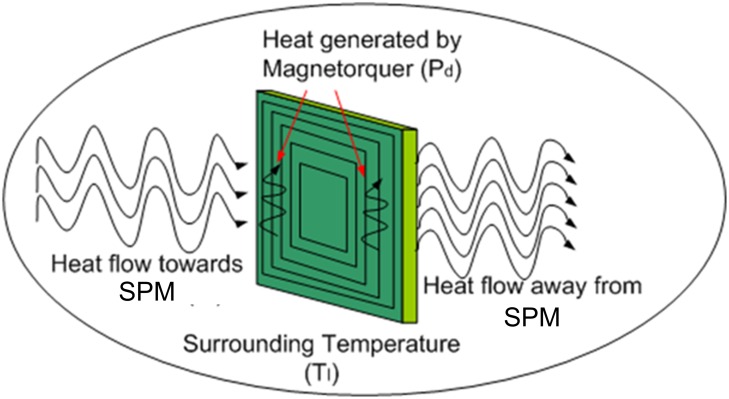
Heat flow through solar panel unit with embedded Air-Coils.

In case of thermal equilibrium, the total emitted power from the solar panel unit to the surroundings (*P*_*o*_) is equal to the electrical power consumed by the coil inside the PCB (*P*_*d*_) and power absorbed from the surroundings (*P*_*I*_) as shown in Fig 11 and given in (7)

$$P_{o}=P_{d}+P_{I}$$ (7)

The power consumed by *M* coils can be inferred from the Stefan-Boltzmann’s law [23];

$$P_{o}=\alpha_{o}\sigma T_{o}{}^{4}S$$ (8)

Where *σ* is the Stefan-Boltzmann constant

*T*_0_ is the solar panel unit surface temperature in Kelvin

*S* is the solar panel unit surface area (both sides)

*α*_0_ is the emissivity of the solar panel material at radiated wavelength

Eqs (7) and (8) result in (9);

$$\alpha_{o}\sigma T_{o}{}^{4}S=P_{d}+P_{I}$$ (9)

In thermal equilibrium condition, no current runs through the coil, *P*_*d*_ = 0, and (9) results in (10);

$$\alpha_{I}\sigma T_{I}{}^{4}S=P_{I}$$ (10)

Where *α*_*I*_ is the emissivity of the solar panel unit surface at the heat absorption wavelength *λ*_*I*_

Inserting *P*_*I*_ value from (10) into (9) resulting in (11).

$$\alpha_{o}\sigma T_{o}{}^{4}S=P_{o}+\alpha_{I}\sigma T_{I}{}^{4}S$$ (11)

In thermal equilibrium, *α*_0_ = *α*_*I*_ = *α*. Rearranging (11) will give a relation between *P*_*o*_ and *T*_*o*_ as given in (12)

$${}_{T_{o}=\sqrt[{4}]{\frac{P_{d}+\alpha \sigma T_{I}{}^{4}S}{\alpha \sigma S}}}$$ (12)

In order to find relation between *P*_*o*_ and *T*_*o*_, it requires emissivity (α), as given in (12).

$$\alpha =\frac{P_{d}}{\sigma S(T_{o}{}^{4}-T_{I}{}^{4})}$$ (13)

The power dissipated (*P*_*d*_) by the air-coil is given by (14), where *I* is the current flowing the air-coil with resistance *R*;

$$P_{d}=I^{2}R$$ (14)

The solar panel unit surface emissivity ‘α’ value which is 0.9 is found through a laboratory experiment [19]. This emissivity value is used for the calculation of *T*_*o*_ in case of different combinations of the Air-Coils discussed in the proceeding section 5.

## 5. Comparison analysis of the embedded Air-Coils various configurations

Different combinations of the Air-Coils are compared on the basis of significant parameters i.e. temperature rise, magnetic moment generated, power dissipated and resultant torque. Current through various configurations of the Air-Coils is controlled by the Air-Coil driver correspondingly related to applied voltage. At a specific voltage, one can acquire the equivalent intended parameters for certain coils arrangements. The satellite power distribution bus (PDB) maximum voltage level is 18V. Therefore, in all measurements the applied voltage is varied from 0V to 18V and the corresponding current flow, power consumed, magnetic moment produced and temperature increase are measured, using various combinations of the Air-Coils. The current through different combinations of the air-coils is measured by using Ohm’s law. The resistance *R* of the single air-coil is 31 ± 3Ω, while four coils in series have 124 ± 3Ω and in four coils in parallel the resistance becomes 8 ± 3Ω. Power dissipated is measured using Eq (14) and temperature is measured by using Eq (12) where all the parameters are known i.e. *T*_*I*_ is the surrounding temperature (25 °C), *P*_*d*_ is the power dissipated by different combinations of the air-coil when the applied voltage is increased from 0V ~ 18V. Where *σ* is the Stefan-Boltzmann constant with value 5.6703× 10^−8^ Wm^−2^K^−4^ and *α* is the solar panel unit surface emissivity which is 0.9, found through a laboratory experiment [18]. Magnetic moment is measured using Eq (1), where *N* is the number of turns of the air-coil, *S* is the coil’s surface area and *I* is the current flowing through the respective combinations of the air-coils. These parameters are given in the design description section 3.

**Fig 12:**
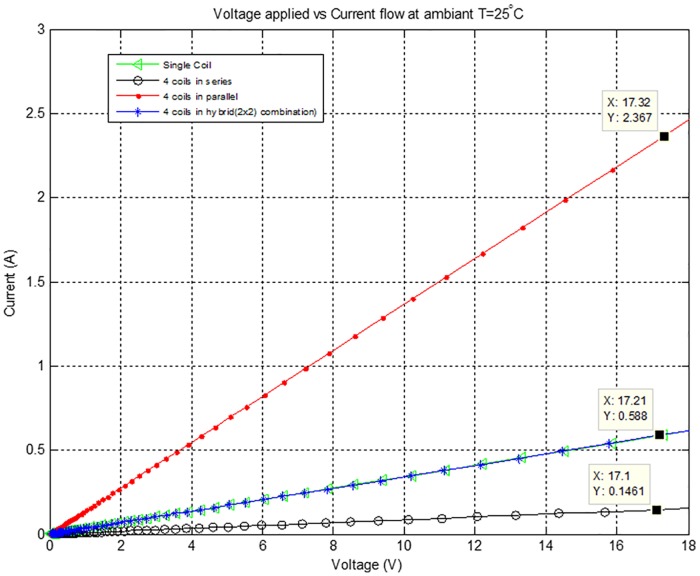
Voltage versus current for different configurations of the Air-Coils.

Fig 12 shows applied voltage versus current characteristics of different configurations of the Air-Coils. At an applied voltage of 17V, the corresponding current drawn by 4 coils connected in series is 0.14A, single coil and 2×2 hybrid combinations draw same current i.e. 0.59A while four coil in parallel draws 2.36A current. The current drawn by different combinations is dependent upon the resistance of the respective coils combination. Four coils in parallel have the lowest, while four coils in series have the highest resistance. This current is also responsible for the power dissipation and overall temperature rise of the SPU module.

**Fig 13:**
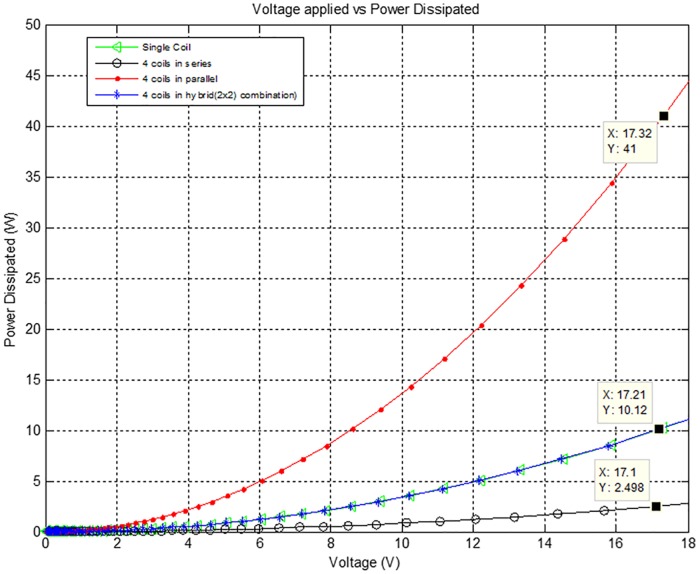
Voltage versus power dissipated for various configurations of the Air-Coils.

Fig 13 shows the applied voltage versus power dissipated characteristics of the Air-Coils various configurations. At an applied voltage of 17V, power dissipated in 4 coils connected in series is 2.5W. At the same applied voltage, 4 coils connected in parallel dissipates 41W power, while in case of single coil and 2×2 hybrid combination, the dissipated power is 11W.

**Fig 14:**
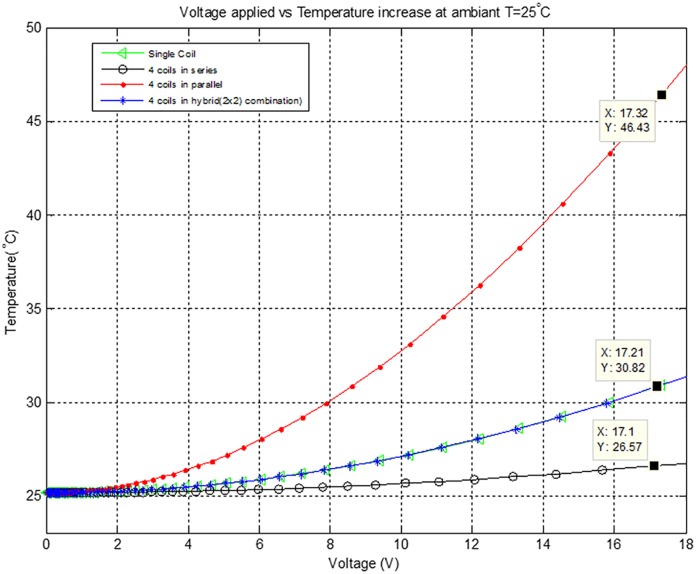
Voltage versus temperature for different configurations of the Air-Coils.

Fig 14 shows applied voltage versus temperature characteristics of different combinations of the Air-Coils. When 17V is applied to the four coils connected in series, the SPM temperature rises to 27°C. In case of single coil and 2×2 hybrid combination, the temperature rises to 31°C, while in case of 4 coils in parallel, the temperature of SPU increases up to 47°C. These results reflect that the designed Air-Coil temperature range is within the constraints of our required microsatellite design’s parameters. Therefore, energizing a specific combination of the Air-Coils, the resulting temperature increase will not damage the solar panel unit with embedded Air-Coils.

**Fig 15:**
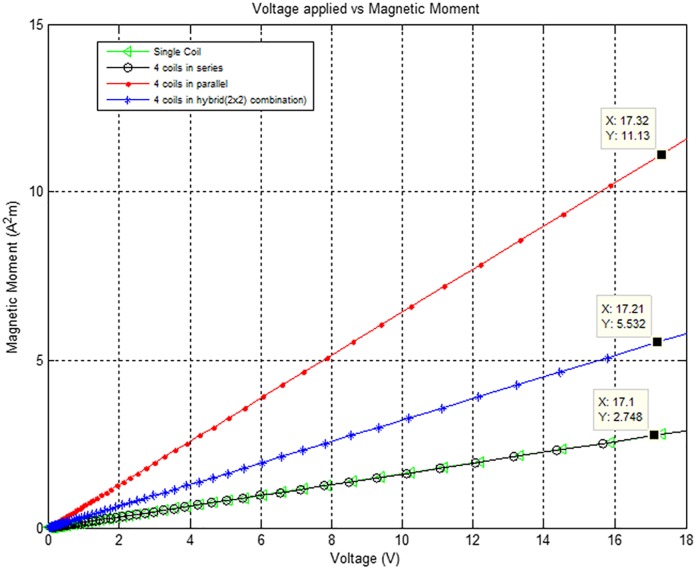
Voltage versus magnetic moment for different configurations of the Air-Coils.

Fig 15 shows applied voltage versus generated magnetic moment for different combinations of the Air-Coils. At an applied voltage of 17V, the magnetic moment generated by single coil and four coils in series is the same i.e. 2.74Am^2^. Hybrid combination generates a magnetic moment of 5.53Am^2^ at 17V applied voltage, while at the same applied voltage, four coils in parallel have 11.13Am^2^ resultant magnetic moment. As already mentioned in section 3, the required magnetic torque for the PNSS-1 is 6Am^2^. To achieve this specific amount of torque, it is required to use four coils in parallel configuration of the Air-Coils at an applied voltage of 8.5V. The same amount of magnetic moment can also be generated from 2×2 hybrid configuration by applying 18V voltage to the Air-Coil.

The generated magnetic moment from series combination is low and fails to meet our requirements. Maximum magnetic moment is yielded from 4 coils connected in parallel. The expected results for such an arrangement includes applied voltage of 17.32V, current drawn is 2.367A, dissipated power of 41W, the generated magnetic moment is 11.13Am^2^ and the observed temperature is 46.43°C. The power dissipation is high in parallel configuration, but when lower voltages are applied the magnetic moment and the rise in temperature can be controlled. These results show that desired amount of magnetic moment for the required microsatellite can be attained by utilizing 4 coils in parallel arrangement.

### 5.1. Time versus temperature rise

Input energy flowing from the surrounding towards the solar panel unit is dependent on the surrounding temperature, emissivity, and surface area and is given by Eq (10). Surface temperature of the solar panel module with respect to power dissipated and heat absorbed from the surrounding is given by Eq (12). Fig 13 gives voltage versus temperature characteristics of the solar panel module with embedded four air-coils in parallel which shows that the maximum temperature rise is 47°C when maximum voltage (18V) is applied. The torque generated by four coils in parallel is 944uNm and will take 202s time for 90° rotation. In order to ensure that the solar panel unit temperature will not increase beyond certain temperature limits even when the magnetorquer coil is kept energized for a longer time (40minutes), a laboratory experiment was conducted. In the experiment the solar module with embedded magnetorquer is placed inside a vacuum chamber. The temperature at normal atmospheric pressure is around 20°C when no voltage is applied, as shown in Fig 15. At time equal to 25.3 minutes the coils are energized by applying voltage of 16.8 V. The amount of current flow and corresponding increase in temperature has been recorded through a data acquisition system. A current of 2.37A start flowing in the Air-Coils with 39.8W power consumption. The temperature started rising and after 140 minutes time it reached a steady state level and there is a negligible increase in temperature as a function of time. At this time the power is disconnected, which resulted in an exponential decrease in temperature.

**Fig 16:**
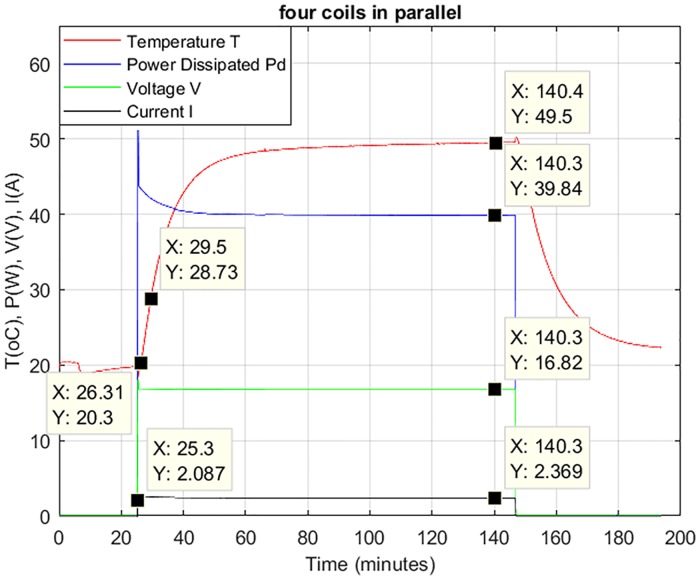
Time vs. temperature rise of the embedded Air-Coil.

As discussed earlier that for 90° rotation the Air-Coil is energized for a time of 202s (i.e. 3.22 minutes). From Fig 16 it is clear that when the coils are energized from time 26.3 minutes to time 28.5 minutes (i.e. for Δt = 3.20 minutes), the temperature increases from 20.3°C to 28.7°C (i.e. ΔT = 8.5°C). The experiment concludes that the temperature rise of the solar panel module, due to activation of the embedded Air-Coils, is very low. Secondly, the Air-Coils are energized at most once a week and will not affect the performance of other subsystems. As the Air-Coils consume high power and when energized, other power hungry subsystems i.e. standard telemetry transmission subsystem, are kept de-energized. The low temperature rise is possible due to the structure material and the dimensions of the solar panel module which result in low thermal resistance and can easily dissipate heat to the surrounding. In the next section (thermal modeling), the thermal resistance of the solar panel module is calculated.

A comparison analysis of the embedded air-coil with CubeSat magnetic rod [24] is given in Table 3. The main parameters of interest for these systems are mass, dimensions, magnetic moment versus power ratio and area occupied. CubeSat magnetic rod has best magnetic moment versus power ratio but is very expensive, heavier and occupies extra space on the satellite is not compatible for PNSS-1. The embedded air-coil discussed in this paper is lighter and requires no extra space on the spacecraft because it is embedded in the PCB four internal layers. Its magnetic moment versus power ratio is also better.

**Table 3:** Comparison of embedded Air-Coil with CubeSat magnetic rod available in the market.

Parameter	Price	Weight (mg)	Dimensions (mm)	Area Occupied Inside the PCB (m^2^)	Power Consumed (W)	Magnetic Moment (Am^2^)	$\frac{D^{2}}{P}\left(\frac{{\left(Am^{2}\right)}^{2}}{W}\right)$.
**CubeSat Magnetic Rod**	€1200	30000	Length = 70 Diameter = 9	External to PCB	0.2	0.2	0.2
**Embedded Air-Coil**	Internal to the PCB (No extra price)	Internal to the PCB (almost weightless)	Internal to the PCB (No space occupation)	0.2025	41	12	3.5

## 6. Thermal modeling of SPM with embedded Air-Coil

In order to maintain spacecraft operational integrity in space thermal environment, thermal modeling is an integral part of the spacecraft subsystem design. In space, heat transfers through conduction only while there is no convection which results in high temperature difference between spacecraft’s surface facing the sun and the surface toward dark. The SPM subsystems absorb solar energy, convert some of the energy into useful electric power while the remaining energy is transmitted to the spacecraft in the form of heat. Some portion of the absorbed heat is dissipated to the cooler opposite face of the spacecraft while the remaining energy is trapped inside the satellite [25]. The trapped heat increases the overall temperature of the spacecraft and affects the performance of the satellite subsystems. The trapped heat which is responsible for the temperature increase depends on the thermal resistance of the SPM. If the SPM PCB material has greater thermal resistance, it results in larger heat accumulation and less heat dissipation to the surrounding [26]. The thermal resistance of the SPM is dependent on the structure material and its dimensions. Therefore, thermal modeling is an essential element in the design process of a solar panel unit as it gives us a complete picture of the heat absorbed and dissipated by the satellite subsystems. In order to minimize the heat accumulation inside the spacecraft, the designer can choose material with lower thermal resistivity and specific dimensions which results in overall lower thermal resistance. Through this technique, the extra subsystems required for heat removal and its effects mitigation can be avoided, resulting in overall spacecraft cost and mass reduction. Thermal resistance is found by using (15) [27, 28];

$$\theta_{th}=\frac{L}{k\times S}$$ (15)

Where *θ* is the thermal resistance, *L* is the length, *K* is the thermal conductivity and *S* is the surface area perpendicular to heat flow.

The unwanted heat power (*P*) delivered to the system is dependent on the solar panel conversion efficiency (*η)*. Greater the value of *η*, more power will be converted to useful electrical power and less will be delivered as unwanted heat to the subsystem components inside the satellite. The value of *P* is given by (16), where ‘*α*’ is the absorption coefficient, *P*_*d*_ is the solar power density and ‘*A*’ is the solar panel area exposed to solar radiations.

$$P=\alpha .p_{d}.A-\alpha .p_{d}.A.\eta .=p_{d}.A.\left(1-\eta \right).\alpha$$ (16)

If thermal resistance of the solar panel material is known, temperature difference ‘*ΔT*’ can be found using (17);

$$\Delta T=P.\theta_{th}$$ (17)

**Fig 17:**
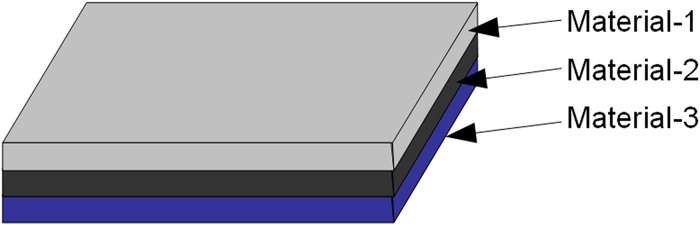
System composed of three different materials.

**Fig 18:**
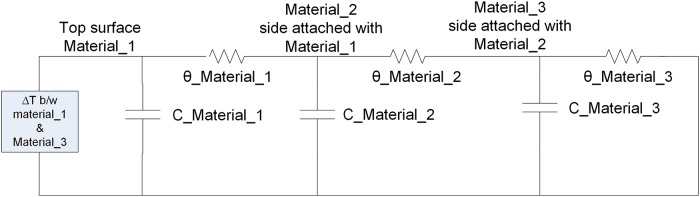
Transient state thermal model.

**Fig 19:**
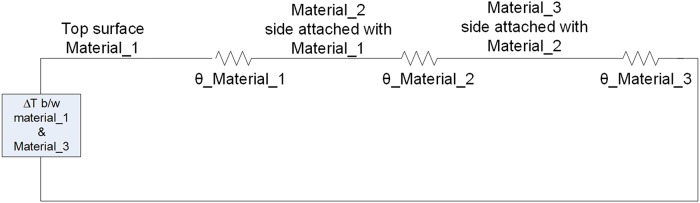
Steady state thermal model.

Suppose a system that is composed on three different materials superimposed on each other as shown in Fig 17, the thermal model can be presented for this structure as a network of capacitors and resistors [29]. It can be modeled in transient or steady state conditions. In transient thermal model, both the thermal capacitance and thermal resistance are taken into account, as shown in Fig 18. In the steady state model, when the temperature and power reaches constant and stable levels, thermal capacitors are fully charged and can be neglected. The steady state model of the structure shown in Fig 17 is given in Fig 19.

In Figs 17 and 18, Δ*T* is the temperature difference between material_1 and material_3.

*θ_material_1* is the thermal resistance of material-1
*θ_material_2* is the thermal resistance of material-2
*θ_material_3* is the thermal resistance of material-3
*C_material_1* is the thermal capacitance of material-1
*C_material_2* is the thermal capacitance of material-2
*C_material_3* is the thermal capacitance of material-3

### 6.1. Thermal resistance

**Fig 20:**
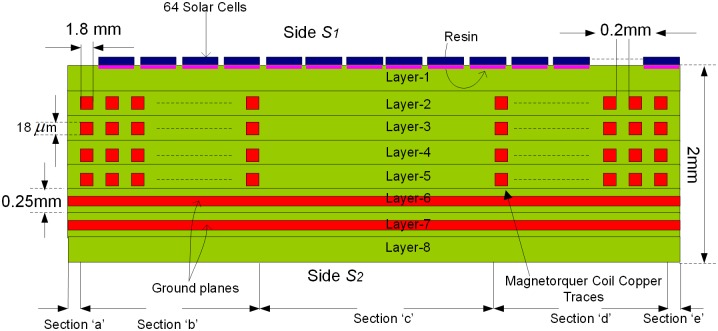
Cross sectional view of SPM.

**Fig 21:**
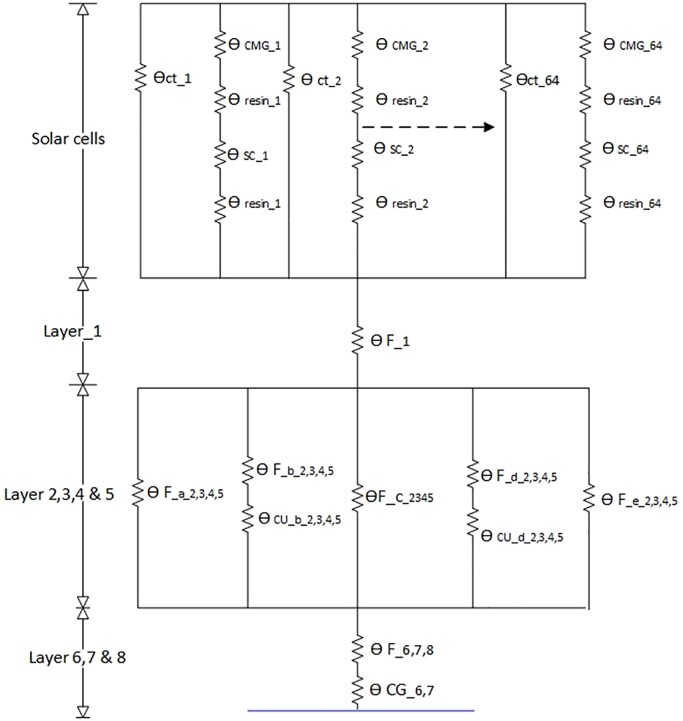
SPM thermal model.

Thermal resistance of SPM is found by utilizing the steady state thermal modeling concept [28]. In steady state thermal modeling, it is assumed that all the capacitors of the network are fully charged and only thermal resistors contribute to the thermal modeling of the system. The internal view of the SPM panel and the corresponding thermal model are shown in Figs 20 and 21 respectively. The SPM panel with embedded Air-Coil under discussion consists of total 8 layers FR4 PCB. The solar cells are mounted on layer 1 by the help of a thermally conductive resin. Layers 2, 3, 4 & 5 are composed of Air-Coil traces embedded inside FR4 material. The internal layers have a total of 260 copper traces (65 in each layer) of certain dimensions while layers 6 & 7 have the ground planes with FR4 material.

In order to achieve a complete thermal model, the solar panel has been divided into various subparts with respect to the type of material. Each part has the corresponding thermal resistance as shown in thermal model of Fig 21. The thermal resistance value is based on the length and width parameters and also material type of the respective part of the PCB. In the comprehensive model, resistance of the solar cell *θ*_*s*_ is in series with its corresponding resin resistance *θ*_*R*_. In thermal resistance depiction, *θ* represents the thermal resistance, *F* denotes FR4, *Cu* reflects copper, the alphabet letters (*a*, *b*, *c*, *d*, *e*) symbolize the respective subsection, and numbers (*1*, *2*, *3*, *4*) denote the relevant layers. For example $\theta_{{F_{a}}_{2,3,4,5}}$ denotes the thermal resistor of FR4 material in subsection ‘*a*’ of layers *2*, *3*, *4 & 5*.

The subscript notations used in thermal model are described in Table 4. The equivalent resistances are calculated by inserting the width, length and conductivity parameters in Eq (15). As an example, how to calculate the resistance values, is given in (18) using (15).

$$\theta_{CT}=\frac{L}{K\times S}=\frac{0.018mm}{355\frac{w}{km}\times (65\times 1.8mm^{2})}$$ (18)

$$\theta_{CMG}=\frac{L}{K\times S}=\frac{0.1mm}{0.8\frac{w}{km}\times (360\times 420mm^{2})}=0.86mk/w$$

**Table 4:** Description of the parameters used in thermal model.

Parameters	Description
*ϴ*_*CT*_	Copper trace thermal resistance
*ϴ*_*CMG*_	Qouptic glass thermal resistance
*ϴ*_*resin*_	Resin thermal resistance
*ϴ*_*SC*_	Solar cell thermal resistance
*ϴ*_*F_1*_	Layer1 FR4 material thermal resistance
*ϴ*_*F_a_2*,*3*,*4*,*5*_	Thermal resistance of FR4 material in section ‘a’ of Layers 2, 3, 4 & 5
*ϴ*_*F_b_2*,*3*,*4*,*5*_	Thermal resistance of FR4 material in section ‘b’ of Layers 2, 3, 4 & 5
*ϴ*_*F_c_2*,*3*,*4*,*5*_	Thermal resistance of FR4 material in section ‘c’ of Layers 2, 3, 4 & 5
*ϴ*_*F_d_2*,*3*,*4*,*5*_	Thermal resistance of FR4 material in section ‘d’ of Layers 2, 3, 4 & 5
*ϴ*_*F_e_2*,*3*,*4*,*5*_	Thermal resistance of FR4 material in section ‘e’ of Layers 2, 3, 4 & 5
*ϴ*_*Cu_b_2*,*3*,*4*,*5*_	Thermal resistance of copper material in section ‘b’ of Layers 2, 3, 4 & 5
*ϴ*_*Cu_d_2*,*3*,*4*,*5*_	Thermal resistance of copper material in section ‘d’ of Layers 2, 3, 4 & 5
*ϴ*_*F_6*,*7*,*8*_	Thermal resistance of FR4 material in Layers 6, 7 & 8
*ϴ*_*CG_7*,*8*_	Thermal resistance of copper ground material in Layers 7 & 8

Mathematical representation of the thermal model of the SPM module shown in Fig 21 is given by (19).

$$\theta_{th}=\frac{\theta_{ct}}{64}||\left(\frac{\theta_{CMG}+2\theta_{re\text{sin}}+\theta_{SC}}{64}\right)+\theta_{F\_1}+\left[\begin{array}{l} \theta_{F\_a\_2,3,4,5}||\left(\theta_{F\_b\_2,3,4,5}+\theta_{Cu\_b\_2,3,4,5}\right)||\theta_{F\_c\_2,3,4,5}||\\ \left(\theta_{F\_d\_2,3,4,5}+\theta_{Cu\_d\_2,3,4,5}\right)||\theta_{F\_e\_2,3,4,5} \end{array}\right]+\theta_{F\_6,7,8}+\theta_{th}$$ (19)

By inserting the length, width and conductivity values of the SPM PCB different sections, as shown in Fig 21, into the mathematical thermal model of (19), one can get the thermal resistance of the SPM module which is 0.02k/W. Thermal resistance value of the SPM module is very small which results in a very low temperature difference between the top and bottom surfaces of the SPM module and will quickly dissipate heat to the cooler side (not exposed to the sun) of the spacecraft. Suppose 1365W/m^2^ of solar power density is impinging on the SPM with dimensions 450mm×470mm, using Eq (17) will result in Δ*T* = 27k between top and bottom of SPM.

## 7. Conclusion

This paper demonstrates that the designed solar panel unit generates sufficient power to drive the microsatellite subsystems and the proposed embedded reconfigurable Air-Coil unit. The designed Air-Coil is incorporated inside the four internal layers of PCB which does not occupy extra space and is almost massless. The coils are copper traces of very small dimensions integrated inside the PCB. Any proposed arrangement of coils discussed in the paper can be used for control and orientation of the microsatellite. This reconfigurable design provides choice in producing any amount of magnetic moment, controlling power consumption and heat generation inside the PCB. Different arrangement of coils have been compared and the most suitable one (4 coils in parallel) is selected according to the mission life, power requirement, heat dissipation and required magnetic moment generation for the maneuverability of microsatellite. The generated magnetic moment is very high i.e. around 12Am^2^, which is enough to rotate a microsatellite by 90° within a time span of 3, 4 minutes. The air-coil is compared with a magnetic rod available in the market on the basis of price, weight, dimensions and magnetic moment generated versus power consumption. The magnetic rod is very expensive with a price of €1200, has a weight around 30g and dipole moment versus power ratio is very low (i.e. 0.2). In comparison the designed air-coil is almost massless, priceless (PCB traces) and dipole moment versus power ratio is very very high (i.e. 3.5). More importantly, thermal analyses have been done in detail to ensure the validity of the proposed embedded Air-Coil and the efficacy of the system operation. Thermal values and resistances show that the heat generation inside the PCB is lower and within the constraints of the required microsatellite design.
